# The Potential Role of the Ketogenic Diet in Serious Mental Illness: Current Evidence, Safety, and Practical Advice

**DOI:** 10.3390/jcm13102819

**Published:** 2024-05-10

**Authors:** Joanna Rog, Zuzanna Wingralek, Katarzyna Nowak, Monika Grudzień, Arkadiusz Grunwald, Agnieszka Banaszek, Hanna Karakula-Juchnowicz

**Affiliations:** 1 Laboratory of Human Metabolism Research, Department of Dietetics, Institute of Human Nutrition Sciences, Warsaw University of Life Sciences (WULS-SGGW), Nowoursynowska 66 Str., 02-787 Warsaw, Poland; 2 1st Department of Psychiatry, Psychotherapy and Early Intervention, Medical University of Lublin, Głuska 1 Str., 20-469 Lublin, Poland; z.wingralek@gmail.com (Z.W.); katarzyna.nowak235@gmail.com (K.N.); monika.grudzien.mg@gmail.com (M.G.); banaszek.agnieszka14@gmail.com (A.B.); hanna.karakula-juchnowicz@umlub.pl (H.K.-J.)

**Keywords:** ketogenic diet, nutritional intervention, bipolar disorder, major depressive disorder, schizophrenia, nutritional psychiatry, psychiatric disorders, mental health, serious mental illness

## Abstract

The ketogenic diet (KD) is a high-fat, low-carbohydrate diet that mimics the physiological state of fasting. The potential therapeutic effects in many chronic conditions have led to the gaining popularity of the KD. The KD has been demonstrated to alleviate inflammation and oxidative stress, modulate the gut microbiota community, and improve metabolic health markers. The modification of these factors has been a potential therapeutic target in serious mental illness (SMI): bipolar disorder, major depressive disorder, and schizophrenia. The number of clinical trials assessing the effect of the KD on SMI is still limited. Preliminary research, predominantly case studies, suggests potential therapeutic effects, including weight gain reduction, improved carbohydrate and lipid metabolism, decrease in disease-related symptoms, increased energy and quality of life, and, in some cases, changes in pharmacotherapy (reduction in number or dosage of medication). However, these findings necessitate further investigation through larger-scale clinical trials. Initiation of the KD should occur in a hospital setting and with strict care of a physician and dietitian due to potential side effects of the diet and the possibility of exacerbating adverse effects of pharmacotherapy. An increasing number of ongoing studies examining the KD’s effect on mental disorders highlights its potential role in the adjunctive treatment of SMI.

## 1. Introduction

The ketogenic diet (KD) is a high-fat, adequate-protein, and low-carbohydrate diet [1]. With the reduced intake of glucose, fat becomes an energy substrate, leading to increased ketogenesis. Oxidation of fatty acids in the mitochondria produces large amounts of energy with acetyl coenzyme A (acetyl-CoA) production. The efficiency of the Krebs cycle is reduced, and the production of mainly three ketone bodies is increased: acetoacetate, acetone, and β-hydroxybutyrate [2,3]. Thus, following the KD leads to intensified production of ketones, which become the main source of energy for the central nervous system [3] and can provide up to 60–70% of the brain’s energy requirements [4,5]. The metabolic state during the KD is described as “nutritional ketosis”. There are many modifications of KD [1]. The classic KD contains 80% fat, dominated by long-chain fatty acids, 15% protein, and 5% carbohydrates [3]. The high-protein KD (Modified Atkins Diet: MAD), known as the Atkins diet, is less restrictive than the traditional KD. The MAD contains 15% carbohydrates with unlimited protein and fat, which makes compliance easier for the patient [3,6]. Meanwhile, reducing long-chain fatty acids and increasing medium-chain fatty acids accelerates triglyceride absorption. This increases the amount of produced ketone bodies per kilocalorie and improves mitochondrial metabolism [1,7,8]. The very low-calorie KD (VLCKD) limits daily carbohydrate intake to 20–50 g or less than 10% of the macronutrients in a 2000 kcal per day [9]. Another modification includes the use of low-glycemic-index products in a high-fat diet [10,11]. In the cyclic KD (CKD), periods of the KD and the high-carbohydrate diet are alternated [12]. A growing number of studies highlight the positive effects of the KD on the composition of the gastrointestinal microbiome [13], mitochondrial activity [14,15,16], neurotransmitter synthesis, and inhibition of neurodegenerative processes [17], as well as modulating oxidative stress and inflammation [18,19,20,21]. At the same time, the increase in the amount of ketone bodies contributes to the “sparing” of glucose. As a result, it can be used to a greater extent in protective antioxidant or glycogenesis processes [22,23,24]. The use of a KD can potentially improve the response to treatment and reduce the symptoms of serious mental illness (SMI): bipolar disorder (BD) [15], schizophrenia (SZ) [25], and major depressive disorder (MDD) [26]. More and more evidence highlights the importance of nutrition in maintaining mental health. Many mechanisms engaged in the pathophysiology of mental illness are affected and modulated by nutrition [27]. Based on these experiences, nutritional psychiatry has been created [28]. Dietary patterns, nutrients, and food products have potential positive effects on mental health outcomes, including the Mediterranean diet; high intakes of vegetables, fruits, and other plant-based products; fermented foods; unsaturated omega-3 fatty acids; vitamin D; zinc, folate, or probiotics; and many others [27]. Mechanisms such as brain glucose hypometabolism, increased oxidative stress and inflammation, dysfunction in neurotransmitter synthesis, and mitochondrial metabolism have been described in the pathogenesis of mental disorders [17,29]. The KD leads to improved carbohydrate and lipid metabolism, which may inhibit the metabolic disturbances that may occur with many antidepressants and antipsychotic medications [15,30]. In 2018, the International Study Group established the KD as an effective nonpharmacologic intervention for epilepsy. The study, published in 2024, suggests that for the protocols aimed at achieving ketosis as a partial mechanism of therapeutic action, the phrase “ketogenic diet therapy” should be used. There are many indications that the KD may become part of the treatment for many conditions beyond neurological diseases [31]. Therefore, the aim of the study is to determine the potential therapeutic effect of KD in SMI.

## 2. Materials and Methods

The studies included in the narrative review were selected from the PubMed, Google Scholar, and Scopus databases from repository inception to 23 March 2024. To collect model and human studies that evaluated the impact of the KD on MDD, BD, and SZ, the following keywords were used: “ketogenic diet”, “ketosis”, “ketone bodies”, “low carbohydrate diet”, “Atkins diet”, “LCHF”, “KLCHF”, “LC/KD”, “serious mental illness”, “psychiatric diseases”, “schizophrenia”, “major depressive disorder”, and “bipolar disorder”. The study selection was as follows: (1) clinical trials, meta-analyses, animal model research, and case reports were included; (2) articles not written in English, conference abstracts only, review articles, duplicated papers, or papers that do not relate to BP, MDD, or SZ were excluded.

## 3. Therapeutic Effect of the Ketogenic Diet in Schizophrenia

### 3.1. Etiopathogenesis and Potential Role of the Ketogenic Diet

Several mechanisms take place in the process of SZ that lead to impaired synaptic communication. The frequent remodeling of synapses and neurons is very energy intensive, while the brain’s main energy substrate is glucose. Glucose is converted into adenosine triphosphate (ATP) through glycolysis in the cytoplasm, the tricarboxylic acid (TCA) cycle, and oxidative phosphorylation in the mitochondria [32]. The greatest part of ATP energy is needed to reverse ion movements that cause postsynaptic responses [33]. Glucose metabolism produces glutamate and gamma-aminobutyric acid (GABA). Deficits in glucose and synaptic energy supply can disrupt communication and cause abnormal brain function and behavior [34]. The dysregulation of systemic glucose metabolism is observed in SZ [35], and transcriptomic, proteomic, and metabolomic studies have repeatedly shown the glycolysis pathway as being disturbed in both the brain and cerebrospinal fluid of patients with SZ [36,37,38]. Chouinard et al. demonstrated abnormal brain bioenergetics in individuals with SZ using 31P magnetic resonance spectroscopy [39]. A variety of glycolysis-related enzymes have been identified to be dysregulated in SZ [38,40] and its translational animal models, including pharmacological and genetic glutamate/NMDA receptor hypofunction models [36,41,42]. In first-onset, antipsychotic-naive patients with SZ, systemic glucose metabolism anomalies can lead to hyperglycemia, decreased glucose tolerance, and increased resistance to insulin [43,44,45]. These findings indicate that a metabolic-based treatment that bypasses damaged glycolytic pathways and impaired mitochondrial activity may have beneficial therapeutic effects [35]. By bypassing glycolysis, providing alternative energy substrates in the form of ketone bodies, and resetting the processes underlying glucose and energy metabolism, the KD positively impacts normalization of brain energy metabolism [46,47,48]. Additionally, it inhibits histone deacetylases and promotes metabolic regulation [35]. The KD improves neuronal function by lowering glutamate toxicity, increasing GABA inhibitory tone, and decreasing reactive oxygen species (ROS) production [49]. The mechanisms of action of the KD also include optimizing mitochondrial metabolism and neurotransmitter function, strengthening neural network stability, and improving oxidative stress and inflammation. The metabolic, neuroprotective, and neurochemical impacts of the KD may give symptomatic relief to people with SZ [17,50].

Moreover, in recent years, the gut microbiota diversity of patients with SZ has been compared to gut microbiota of healthy individuals. In comparison to the healthy gut, facultative anaerobic bacteria such as *Lactobacillus fermentum*, *Alkaliphilus oremlandii*, *Cronobacter sakazakii*/*turicensis*, and *Enterococcus faecium* were identified among individuals with SZ [51]. Authors suggest that a personalized and targeted modulation of intestinal microbial diversity by prebiotics (non-digestible fiber) might be a treatment option for management of SZ [52]. The KD considerably impacts the variety and count of the gut microbiome, which is linked to reduced blood glucose levels and increased blood ketone levels [53].

**Figure 1:**
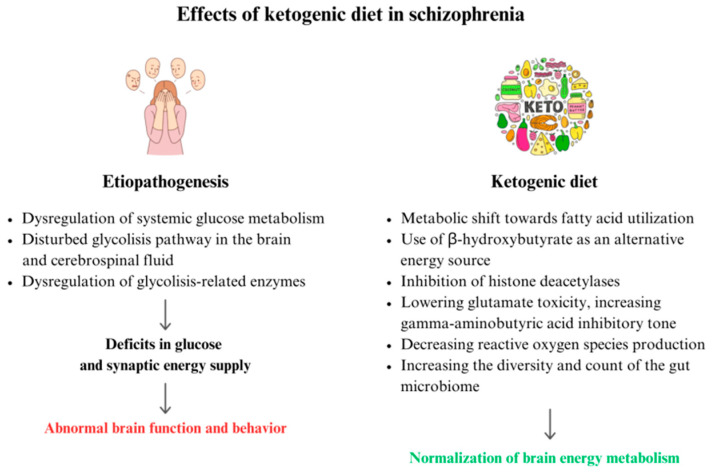
Possible effects of a ketogenic diet therapy in schizophrenia.

The etiopathogenesis of SZ and the potential role of the ketogenic diet In its treatment are summarized in Figure 1. An overlapping mechanism of the pathogenesis of SMI and the therapeutic mechanism(s) of the KD on SMI should be considered.

### 3.2. Animal Model Studies

In 2015, Kraeuter et al. showed for the first time that a KD can regulate abnormal behaviors in an animal model of SZ. In mice, three weeks of KD prevented agitation, stereotypy, and impaired sociability and working memory caused by an acute NMDA receptor hypofunction. These behaviors are comparable to the positive, negative, and cognitive symptoms of SZ [35]. KD successfully reestablished impaired hippocampal inhibitory circuits involved in auditory sensory gating in DBA/2 mice, a model applicable to SZ [49]. Authors applied an evolutionary-conserved schizophrenia-like behavioral endophenotype, impaired sensorimotor gating, as measured by prepulse inhibition of startle (PPI) [35]. Male C57BL/6 mice were fed a KD for seven weeks and tested for PPI at 3 and 7 weeks, with and without a significant digestible energy deficit. They found that the KD successfully prevented MK-801-induced PPI impairments at 3 and 7 weeks, regardless of the presence or absence of a digestible energy deficit. Moreover, there was no link between PPI and body weight fluctuations. The results support the therapeutic effect related to the state of ketosis and not energy restriction in SZ [35]. KD-fed mice demonstrated metabolic adaptation by body weight reduction, higher β-hydroxybutyrate levels, and lower glucose levels. This study did not explore the potential mechanisms of action. However, the authors claim that the KD may help normalize pathophysiological processes in SZ in several ways. Another study investigated how the KD affects hippocampal P20/N40 gating in DBA/2 mice [49]. The animals with the greatest ketone levels exhibited the lowest P20/N40 gating ratios. The KD appears to successfully target sensory gating deficiencies, making it a promising subject for further research in SZ [54]. Antipsychotic medications, such as olanzapine (OLZ), are used to treat schizophrenia and a rising spectrum of other “off-label” diseases. A single dose of OLZ generates significant blood glucose increases within minutes of therapy [55,56,57]. According to a study by Shamshoum et al., fasting or short-term ingestion of a KD protects against OLZ-induced hyperglycemia, regardless of changes in whole-body activity of insulin, and is associated with a reduced rise in serum glucagon [54,58,59,60]. However, rapidly increasing circulating ketone body concentrations with β-hydroxybutyrate or oral ketone esters did not replicate the effects of fasting or the KD. Overall, data indicate that fasting and short-term KD intake can protect against acute AP-induced changes in glucose homeostasis, whereas interventions that enhance circulating ketone bodies do not have the same protective benefits [54].

### 3.3. Clinical Trials and Case Studies

In 1965, researchers noticed numerous variances and/or abnormalities in the carbohydrate metabolism of patients with SZ. They hypothesized that improving glucose metabolism might alleviate symptoms associated with disease. Pacheco et al. conducted a pilot study since the KD would possibly mimic those effects and could be administered safely to patients [61]. Despite the small number of participants, they observed improvements in positive and negative symptoms of SZ in the examined group of patients. Sethi et al. undertook a four-month pilot trial to see how a KD affected individuals with SZ and BD with co-occurring metabolic abnormalities [62]. Individuals who followed the program lost body weight (12%) and reduced their body mass index (BMI) (12%), waist circumference (13%), and visceral adipose tissue (36%). A 27% decrease in the homeostasis model assessment-estimated insulin resistance (HOMA-IR) and a 25% drop in triglycerides were observed. In individuals with SZ, the severity of symptoms was reduced (32% drop in Brief Psychiatric Rating Scale scores). Moreover, a case report by Palmer et al. presents two patients with SZ treated with the KD for 5 and 12 years [63]. The first patient, an 82-year-old female, saw a significant decrease in psychotic symptoms after 2 weeks of following the KD. During the next few months, she decided to discontinue all of her medications. Her mood improved considerably, and she stopped having suicidal thoughts. Her hallucinations subsided totally. The second patient was a 39-year-old female with a history of depression, anxiety, anorexia nervosa, hallucinations, and psychosis. During her treatment, she took several drugs, including haloperidol, clozapine, ziprasidone, risperidone, quetiapine, aripiprazole, olanzapine, sertraline, paroxetine, citalopram, fluoxetine, duloxetine, and venlafaxine. Within one month of the KD, she experienced total clearance of psychotic symptoms. She was taken off haldol-decanoate after a year of treatment and has been free of psychotic symptoms for the past five years without antipsychotic medication. The patient remains on a KD [63]. Kraft et al. reported the unexpected remission of long-standing SZ symptoms in a 70-year-old female patient after beginning a KD [64]. She experienced both auditory and visual hallucinations, which have appeared since the age of seven. The hallucinations stopped after 19 days despite no changes in her medicine. Over 12 months, the patient has maintained a low-carbohydrate KD without a recurrence of symptoms, and her excessive weight has been reduced.

The studies describing the therapeutic effect of the ketogenic diet in schizophrenia are presented in Table 1.

## 4. Therapeutic Effect of the Ketogenic Diet in Depression

### 4.1. Etiopathogenesis and Potential Role of the Ketogenic Diet

Brain regions such as the nucleus accumbens (NAc), medial prefrontal cortex (mPFC), and lateral habenula (LHb) are involved in the development of depressive disorders [65]. MDD leads to chronic low-grade inflammation in the body. Microglia are activated and, as a consequence, there are increased levels of interleukin 6 (IL-6), interleukin 8 (IL-8), interleukin 12 (IL-12), and tumor necrosis factor-α (TNF-α) in the cerebrospinal fluid [66,67], and increased translocator protein, a marker of central inflammation, in the temporal cortex and anterior cingulate cortex of the brain [68,69]. The KD and other types of diets based on restricted carbohydrate intake may prevent the occurrence of MDD, reduce depression symptoms, cause a mood improvement, and lower the risk of cognitive impairment [70]. As previously mentioned, the KD can positively influence the gut microbiota composition. The intestinal microbiota disturbances are intensified and can cause a chronic low-grade inflammatory process, which influences a more severe course of MDD and treatment resistance [66,71,72]. The KD induces ketolytic metabolism, which can lead to increased oxidative phosphorylation with a shift in the glutamate–aspartate aminotransferase balance. As a result, there is an increase in adenosine and GABA activity [73]. In contrast, reduced GABA levels and dysfunction of the GABA-ergic system are often described in MDD [74]. The KD improves the function of uncoupling proteins (UCPs) in the mitochondria, indirectly reducing oxidative stress and the production of ROS [14]. The mechanisms described above result in a reduction in low-grade inflammation [14,73]. Clinical studies have shown that the KD can also affect dopamine, serotonin, and glutamate, which are neurotransmitters that are important in the pathogenesis of MDD, according to monoaminergic theory [75,76]. Huang et al. demonstrated that β-hydroxybutyrate interacts with microglia at the cellular and molecular levels, improving neural plasticity and modulating depressive symptoms [20].

**Figure 2:**
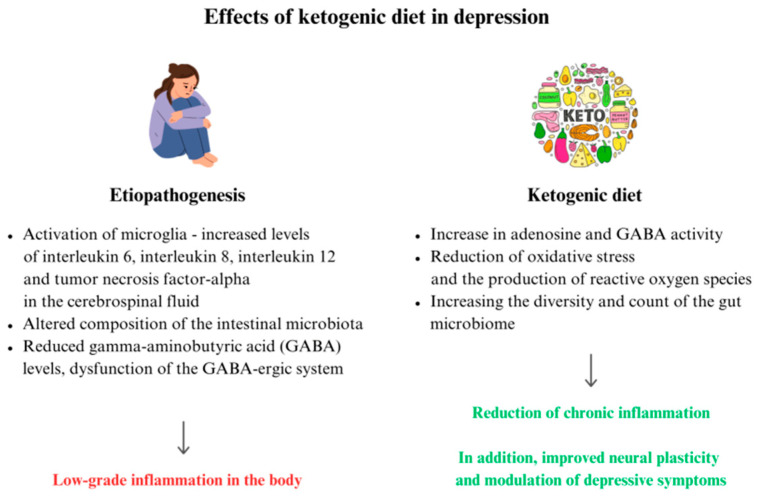
Possible effects of a ketogenic diet therapy in depression.

The etiopathogenesis of MDD and the potential role of the KD in its treatment are summarized in Figure 2. An overlapping mechanism of the pathogenesis of SMI and the therapeutic mechanism(s) of KD on SMI should be considered.

### 4.2. Animal Model Studies

In the animal study conducted by Murphy et al., the KD was related to higher blood β-hydroxybutyrate concentrations and a shorter period of immobility. The greater reactivity indicates that the KD may have similar effects to antidepressants [77]. In another study, susceptibility to depressive and anxiety states was significantly reduced, while physical activity was increased after exposure to a KD in prenatal life. Exposure to the KD was related to a 1.39% reduction in the hypothalamus, a 4.77% reduction in the corpus callosum, and a 4.8% increase in cerebellar volume. Thus, the use of the KD in prenatal life may positively influence neuroanatomical brain and behavioral changes and reduce the risk of depressive symptoms in later adult life [78]. Guan et al. observed that the KD may decrease neuronal excitability in the lateral habenula (LHb), a brain region responsible for the development of MDD. At the same time, in MDD, there is a reduction in the protein level of innate immune receptor Trem2 in the LHb, leading to activation of a microglia and inflammatory response [65]. An additional positive effect of the reduction of depressive and anxiety symptoms can be achieved by physical exercise with the application of the KD. After six weeks of the KD in mice, glucose, insulin, and the LDL/HDL ratio decreased, and β-hydroxybutyrate increased. After the nutritional intervention, the animals displayed fewer anxiety and depressive behaviors [79]. Furthermore, Kasprowska-Liśkiewicz et al. proved that the KD increased motor activity and reduced anxiety in rodents. At the same time, the rats’ social interest increased [80]. The study assessing the type of fatty acids used in the KD showed a reduction in depressive behavior in animals, regardless of whether they were fed long-chain triglyceride or medium-chain triglyceride fatty acids [81].

### 4.3. Clinical Trials and Case Studies

The limitations of pharmacotherapy for MDD determine the need to explore other possible interventions to reduce the severity of the illness [82]. The KD could improve health-related quality of life, including mental health, after 24 weeks in overweight volunteers. However, the study participants were not diagnosed with MDD [83]. Cox et al. described the case of a woman with uncontrolled type 2 diabetes, MDD, hypertension, and dyslipidemia who followed the KD for 12 weeks under medical supervision. She was chronically taking the selective serotonin reuptake inhibitors, lisinopril and glipizide. After the intervention, a significant reduction in the severity of MDD symptoms and an improvement in metabolic parameters such as glycated hemoglobin (HbA1c) and fasting blood glucose were observed. In improving the prognosis of patients with co-occurring MDD and type 2 diabetes, it may be essential to integrate approaches including various changes, e.g., the KD, nutritional education, and physical activity [83]. Danan et al. conducted a one-year analysis of poorly controlled symptoms (despite intensive pharmacological treatment) of severe mental illness, including MDD, following the KD instead of the usual hospital diet. A reduction in MDD symptoms was demonstrated in all of the examined patients according to the Hamilton Depression Rating Scale (HAM-D) scale (mean score decreased from 25.4 to 7.7) and the Montgomery–Åsberg Depression Rating Scale (MADRS) scale (mean score decreased from 29.6 to 10.1). Improvements were shown in metabolic parameters such as BMI, blood pressure, blood concentration of fasting glucose, HbA1c, gamma-glutamine transferase (GGT), alanine aminotransferase, aspartate aminotransferase, total cholesterol, and triglycerides. Good tolerance of the KD was described in the vast majority of patients [82]. In a study conducted by Ohio University, improvements in MDD symptoms were described in 262 people with co-occurring type 2 diabetes after 10 weeks of following the KD [84]. Additionally, in a young woman (21 years) with a co-occurrence of mood disorders with obesity, hypertension, and Turner syndrome, a positive effect of the KD was shown. A depressed mood led to self-harm, disrupted daily rhythms, reduced ability to concentrate, and high suicide risk. After four weeks of application of the KD, decreases in body weight (of 11.5 kg) and BMI (to class II obesity from class III) were observed, as well as stabilization of mood, reduction in anxiety and normalization of daily rhythm. The severity of MDD was described as moderate compared to severe when starting the diet, and the patient did not report suicidal thoughts [74]. The number of registered protocols of studies of the KD in MDD is still increasing. The outcomes of ongoing trials are various, including in terms of MDD symptoms, laboratory tests, and the brain’s electrical activity [85,86]. The studies describing the therapeutic effect of the ketogenic diet in depression are presented in Table 2.

## 5. Therapeutic Effect of the Ketogenic Diet in Bipolar Disorder

### 5.1. Etiopathogenesis and Potential Role of the Ketogenic Diet

**Figure 3:**
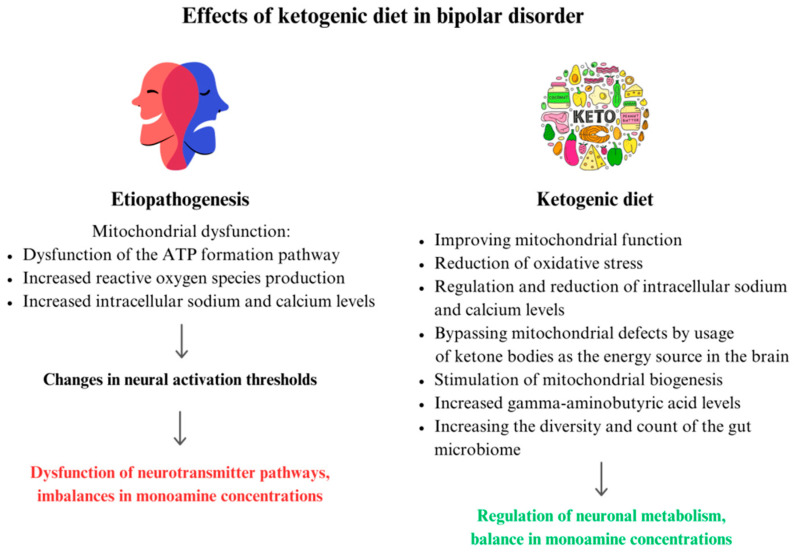
Possible effects of ketogenic diet therapy in bipolar disorder.

BD is characterized by recurrent episodes of manic and depressive states, with transient episodes of euthymia (neutral mood) [87]. A growing body of evidence supports the idea that mitochondrial dysfunction may be an underlying feature of BD [88,89,90]. Abnormal mitochondrial function leads to reduced energy production associated with more cells undergoing apoptosis, increased ROS, and excessive excitability [91,92,93]. There is increasing evidence for the concept of oxidative stress as an underlying mechanism in BD [88,94]. Among patients with BD, elevated intracellular calcium levels are observed regardless of disease phase [95]. Calcium homeostasis, one of the main factors determining apoptosis, is regulated by mitochondria [96,97,98]. Dysfunction of the ATP formation pathway is also a potential factor in the development of BD [99]. Changes in ATP levels affect the timing of neurotransmitter release and the transition of neurons to excitatory or inhibitory states, and may contribute to the manic and depressive states in BD [100]. Reduced Na+/K+ ATPase activity and increased intracellular sodium levels have been observed among individuals with BD. Under conditions of Na+/K+ ATPase hypofunctionality, sodium accumulates in neurons and alters the resting potential, resulting in altered neuronal excitability [101]. Imbalances in monoamine concentrations may affect behavior and emotions [102]. The KD, which changes the way the organism uses energy, appears to be a promising therapeutic approach for BD. Numerous literature reviews provide evidence that the KD can affect various metabolic and biochemical aspects of BD especially related to mitochondrial function [17]. According to Campbell et al. [103], the KD is able to alleviate the symptoms of BD as a result of changing the main energy source in the brain from glucose to ketone bodies, in effect helping to bypass existing mitochondrial defects and limit further damage to these structures. Ketosis stimulates mitochondrial biogenesis, improves brain metabolism, acts as a neuroprotector, and promotes glutathione synthesis [64,104,105]. Data available in the literature show that lactate levels are consistently elevated and, at the same time, are one of the biomarkers most altered among patients with BD [106]. Research proves qualitative and quantitative changes in intestinal microbiota among patients with BD compared to healthy individuals, suggesting that an imbalance in microbiome composition and function may affect mental health through the gut–brain axis [107,108]. Oxidative stress- and inflammation-inducing *Flavonifractor* bacteria have been linked to BD [109]. Dickerson et al. demonstrated that probiotic treatment of people with BD contributed to shorter patient hospitalizations [110]. Studies show that the KD significantly alters the diversity and count of the intestinal microbiome (towards potentially beneficial taxa) [53,71]. This may be linked to reduced intake of carbohydrates (including refined sugars, which directly affect the functionality of intestinal microbiota); this is synonymous with reduced polysaccharides, from which the bacteria derive their energy [111]. The etiopathogenesis of bipolar disorder and the potential role of the ketogenic diet in its treatment are summarized in Figure 3. An overlapping mechanism of the pathogenesis of SMI and the therapeutic mechanism(s) of KD on SMI should be considered.

### 5.2. Animal Model Studies

Unfortunately, BD remains challenging to model in animal experiments. Scientists have noted during human and animal studies that neurotransmitter imbalances may contribute to the development of BD. Clinical observations are the source of reports that changes in dopamine (DA) levels are present in episodes of BD. Manic episodes are associated with hyperdopaminergic and depressive episodes with reduced dopaminergic transmission [103], and the KD contributes to altered levels of monoamine metabolites, including a significant reduction in dopamine [75]. The KD leads to an increase in *Lactobacillus* and *Akkermansia*, while decreasing potentially pro-inflammatory bacteria from the genii *Desulfovibrio* and *Turicibacter*. This has been confirmed in rodent studies [53]. This information may be clinically useful due to the fact that *Akkermansia*-containing probiotics showed antidepressant properties in an animal model of stress, which also indicates their potential role in antidepressant effects in patients with BD [112].

### 5.3. Clinical Trials and Case Studies

Danan et al. [82] conducted a retrospective analysis with a total of 31 participants, 13 of whom suffered from BD type two. Patients were admitted to a psychiatric hospital and put on a KD for up to 248 days. Symptoms were poorly controlled despite the intensification of psychiatric treatment. After the dietary intervention, a significant change in the patients’ well-being and reduced severity of mood symptoms was observed. Additionally, a reduction of 1 point on the Clinical Global Impressions–Severity Scale is considered a minimal clinically relevant difference; in the study, the average decreased by 2.8 [113]. A non-randomized, interventional pilot study confirmed the feasibility and safety of introducing a KD for three months among individuals with BD [113]. Phelps et al. presented the cases of two women with BD II who maintained a KD for an extended period of 2 and 3 years, respectively. In both cases, there was an improvement in mood stability, and the effects were more significant than those achieved through pharmacotherapy. The lamotrigine used by the first patient could not provide reliable symptom control. The second patient had severe side effects from the medication used, including an increased frequency of suicidal thoughts. Both women tolerated the dietary intervention well and reported a marked improvement in well-being as a result of being in ketosis. In neither case were there any significant side effects. The researchers additionally believe that regularly maintaining urinary ketone body levels of at least 5 mg/dL helped control BD symptoms [114]. Another paper describes the case of a patient whose mood stabilization was observed after following a KD for about two years and cyclical one-day fasting (to increase the intensity of ketosis). The complete absence of depressive episodes had accompanied the patient for eight years, and the patient decided to withdraw from quetiapine [115]. Campbell et al. conducted an observational analytical study of comments in online forums regarding the effects of dietary interventions (KD, omega-3 enriched, or vegetarian) on mental health in 274 patients with BD. Eighty-five people reported beneficial effects associated with the KD: improved mood stability (*n* = 55); fewer episodes of depression (*n* = 35); improved clarity of thought and speech (*n* = 24); increased energy levels (*n* = 22); fewer anxiety/panic attacks (*n* = 17); fewer episodes of mania (*n* = 1); improved sleep quality (*n* = 7); improved control of activities (*n* = 7); and improved memory (*n* = 2). The duration of improvement in mood stability was often reported to be months or years (the longest period was 8 years). It should be borne in mind that there are many limitations to the study due to its retrospective nature [116]. A pilot study involving 27 patients with BD found that following a KD for 6–8 weeks leads to decreased lability and lactate. Authors found a positive relationship between ketone levels and ratings of momentary mood energy, and a negative correlation between ketone levels and impulsivity and anxiety [117]. Sethi et al. conducted a 4-month pilot study involving 16 people with BD. After dietary intervention, increased life satisfaction and better sleep quality were observed. It was shown that 69% of participants with BD experienced improvements in the severity of mental illness [62]. The studies describing the therapeutic effect of ketogenic diet in bipolar disorder are presented in Table 3.

Currently, there are still ongoing studies that are likely to provide more detailed information on the importance of the ketogenic diet among patients with BD and other psychiatric disorders. Ongoing clinical trials exploring the role of the KD in psychiatric disorders are presented in Table 4.

## 6. Health Risks Associated with the Use of the Ketogenic Diet

Despite many positive aspects of using the KD, some disadvantages and risks must be mentioned. Some patients experience one or more symptoms during the early adaptation of the KD [82,127]. Among the most commonly reported early side effects of the KD, patients present “ketone flu” (a set of temporary, general malaise symptoms), headache, nausea, fatigue, weakness, gastrointestinal symptoms, and change in heart rate. However, after a few weeks following the KD, the side effects pass in most patients [127]. For severe gastrointestinal symptoms, the treatment may include H2 blockers, proton pump inhibitors, increased fluid intake, and the addition of fiber-containing products [128]. If the balance of the diet is not adapted to the individual requirements, it can cause complications such as dehydration, electrolyte disturbances, hypercholesterolemia, and nutritional deficiencies [129,130]. A very restrictive KD with a limited supply of fruit, whole grains, vegetables, or legumes can lead to nutritional deficiencies in folic acid, thiamin, vitamin A, vitamin E, vitamin B6, calcium, magnesium, and potassium levels, as well as an inadequate intake of fiber and protein and an excess intake of fats [129,131]. Therefore, a dietician should be consulted first to determine the appropriate intake of nutrients. Long-term adherence to the KD can be problematic for the patient due to the need for lifestyle changes. However, proper education and explanation of the mechanisms and clinical effects motivate patients to adhere to the KD [131,132]. The most common long-term side effects include hyperlipidemia, hypertriglyceridemia, renal stones, cardiovascular complications, mineral and vitamin deficiencies, and electrolyte imbalance [133,134,135]. Monitoring of specific laboratory parameters while following the KD is required. In particular, monitoring of renal function should be undertaken, including urinalysis: albumin, creatinine, calcium/creatinine ratio, and blood creatinine to estimate the glomerular filtration rate (eGFR), due to the risk of developing nephrolithiasis and chronic kidney disease [128,131,132]. Adequate hydration and monitoring of electrolyte blood concentration are also important. The use of citrates to normalize urine pH may be considered [127]. Taking into consideration the increased cardiovascular risk of patients with SMI and the high content of fat in the KD, determining the lipid profile (total cholesterol, LDL and HDL, triglyceride concentrations), and liver parameters, and performing an ECG or ECHO, are recommended [128]. The KD is not recommended in patients with liver failure, chronic pancreatitis and in patients with diabetes on insulin treatment or with recurrence of severe hypoglycemia [132]. The KD can lead to chronic malnutrition of the body, reduced bone density, and menstrual cycle disorders with the co-occurrence of the diseases as mentioned above [135,136,137]. The KD should not be recommended for women who are pregnant or planning to become pregnant. A study by Desrosiers et al. found that low-carbohydrate diets were more likely to result in folic acid deficiency, increasing the risk of having a baby with a neural tube defect [138]. Despite some limitations, the KD shows a number of clinical benefits. It reduces the risks of obesity, metabolic syndrome, and type 2 diabetes, disorders that often co-occur with SMI [139]. The KD should be implemented in hospital conditions to ensure constant supervision, physician care, and patient safety [140,141].

## 7. A Practical Guide to Using the KD in Psychiatric Disorders

The choice to use the KD depends on many patient-related aspects and it should always be applied with high-quality care of a physician and dietitian [142,143]. The potential side effects related to some medications and multimorbidity should be considered when planning the diet. The KD is high in fat, so lipid disturbances could occur, especially in patients receiving atypical antipsychotics [144,145,146]. Ensuring a healthy dietary fatty acid profile (mono- and polyunsaturated fatty acids, while avoiding saturated and trans-unsaturated fatty acids) and cooking techniques (cooking, grilling, and baking instead of frying) minimizes the risk of hypercholesterolemia. The carbohydrate restriction leads to a low amount of fiber in the diet and could lead to constipation [147]. The fiber also positively affects lipid metabolism. The anticholinergic effect of some medications (clozapine, olanzapine, phenothiazine derivatives, or tricyclic antidepressants) could cause severe constipation [148]. Fiber-rich vegetables with low amounts of carbohydrates should be chosen. Monitoring the daily amount of fluid intake may be helpful. The anti-constipation effect of physical activity should also be taken into account. However, in implementing the KD and an inadequate intake of energy, physical activity could be harmful. Supplementation of omega-3 fatty acids, recommended in SMI, can stimulate intestinal peristalsis. Another problem of the KD is the inadequate amount of calcium [149]. Vitamin D supplementation, according to guidelines, allows for increased calcium absorption up to 30–40% [150]. Products with high calcium content and recommended in the KD should be implemented. One of the examples of a healthy, balanced ketogenic diet is its Mediterranean version. Its main principles include using olive oil and vegetable fat sources, limiting products rich in saturated fatty acids, using high-biological-value protein sources, and including seasonal fruits and vegetables with every meal [151].

Despite the many potential advantages of implementing the KD, patients could face many difficulties [152]. Longer-term adherence to the diet in patients with psychiatric disorders remains uncertain. In pilot studies, adherence is reported at 65–80% [113]. The application of the KD is challenging due to several reasons. Developing strategies to improve patient compliance might increase the number of individuals benefiting from the KD [152]. According to studies, low compliance results from the inability to contact the nutritionist, physiological disturbances affecting blood laboratory tests, unresponsiveness to diet, or insufficient knowledge about the KD [113,152].

Available scientific data show patients with mental disorders have poor nutrition and mental disorders negatively influence dietary intake. Patients’ symptoms could have a negative impact on compliance, such as impulsivity, apathy, reduced appetite, food cravings, and binge eating [153]. Psychological support and behavioral components to increase the likelihood of compliance are essential [154]. Intervention should address three factors influencing adherence: capability, opportunity, and motivation. Additional psychological techniques, such as integrated motivational interviewing and cognitive behavior therapy, can lead to improvements in diet adherence [154]. In pilot studies, support included problem-solving [154], identifying and managing side effects, personalizing dietary prescriptions, and being supervised by specialists.

Many medications are associated with substantial increases in appetite and uncontrolled food intake [155]. Antipsychotic medication affects dietary intake and eating behaviours, and patients may fail to implement any dietary restrictions. The KD is demanding and highly restricted, which may discourage patients from implementing it, especially with problems with overeating. Many patients report decreasing satiety and cravings for non-nutritious foods with high sugar and fat content. Some health practitioners might not believe in the positive effect of diet implementation, which reduces the patient’s motivation. According to a study, less than 5% of patients with mental health problems receive lifestyle advice, despite 80% of them expressing a desire for lifestyle medicine from their general practitioner [155]. In most mental health centers, the availability of dietitians is limited. The lack of a specialist makes it challenging to implement and monitor patients’ progress and discuss and solve problems encountered during therapy. The increased possibility of implementing dietary recommendations will allow us to obtain more data about the potential incorporation of a diet in clinical practice in the treatment of mental disorders.

The practical advice is presented in Table 5.

## 8. Strengths and Limitations

Our review has several strengths and limitations to consider. Firstly, the analysis included the manuscripts indexed in one of the three most popular databases, and strictly defined keywords for relevant literature obtained were used. Apart from the completed studies, the authors included ongoing studies in the review to better determine the current concepts and proposals for the potential use of the KD in patients with mental diseases. The practical advice for psychiatrists and patients was proposed based on literature analysis. Despite the growing interest in the diet, studies discussing practical guidance for implementing and monitoring diet therapy are still limited. Due to a lack of guidelines, clinical application is elusive and, for some practitioners, a major challenge.

The studies included in this review article were mainly pilot studies or case reports with poor quality and strength of evidence. However, the number of clinical trials is still too scarce to provide significant and reliable findings. The authors used different tools to assess the effect of the KD on patients’ health and examined different outcomes. We could only analyze articles in English and the selection of studies may reflect a publication bias, as studies with positive outcomes are more likely to be published. There is a lack of evidence about long-term studies investigating the KD in patients with mental disorders. The longstanding consequences of the KD in individuals with a high risk of somatic complications are still not determined.

## 9. Conclusions

The KD shows promise as providing benefits to patients with many serious diseases. However, regarding SMI, the benefits of the KD remain inconclusive at this time. The currently available data highlight the potential therapeutic role and benefits of the KD in SMI. The animal model studies demonstrate the ability of the KD to modulate many pathological processes connected with SZ, MDD, and BD, including disruptions in carbohydrate metabolic pathways, altered neurotransmission, changes in intestinal microbiota composition, mitochondrial dysfunction, inflammation, and oxidative stress. The data from case studies and a few studies with unsatisfactory quality confirm the positive effect of the KD on MDD, BD, and SZ symptoms, including changes in pharmacotherapy (reduction in dosage or complete withdrawal of medication), in some cases. Additionally, following the KD reduces the comorbid symptoms of patients. The number of clinical trials assessing the potential role of the KD in SMI is still limited. However, the number of ongoing studies indicates the therapeutic potential of the KD, and implementation of this results of these trials will enable the verification of the hypothesis.

## Figures and Tables

**Table 1:** Studies describing therapeutic effects of the ketogenic diet in schizophrenia.

Author(s), Year	Study Design	Number of Participants (Intervention/Control)	Range Age of Participants (Years)	Dietary Intervention/ Assessment of Ketosis	Control Intervention	Duration	Outcome Measures	Main Findings
Pacheco et al., 1965 [61]	Pilot study	I: 10 (F)	19–63	KD, lack of detailed information	none	2 weeks	A nursing checklist for ward behavior ratings, The Minimal Social Behavior Scale, The Beckomberga Rating Scale for the S-Factor	↓symptomatology, ↑symptomatology after discontinuing the KD
Kraft et al., 2009 [64]	Case study	I: 1 (F)	70	gluten and low-carbohydrate KD, (<20 g carbohydrates per day), ketosis was not confirmed	none	1 year	Patients’ and physicians’ observations	↓symptomatology (visual and auditory hallucinations), ↓body weight, ↑energy level
Palmer et al., 2019 [63]	Two case studies	I: 2 (F)	82 and 39	KD, lack of detailed information	none	5 years and 12 years	Patients’ and physicians’ observations	↓symptomatology, ↓body weight, ↓the amount of taken medications
Sethi et al., 2024 [62]	Pilot study	I: 5	18–75	KD, 10% carbohydrate, 30% protein, 60% fat; ≥5040 kJ, <20 g carbohydrates per day; blood ketone meter at least once a week	none	4 months	Generalized Anxiety Disorder (GAD–7), Patient Health Questionnaire Depression Scale (PHQ–9), Pittsburgh Sleep Quality Index (PSQI), Clinical Mood Monitoring Forms (CMF), Clinical Global Impression–Schizophrenia (CGI–SCH) Scale, Global Assessment of Functioning (GAF), Manchester Short Assessment of Quality of Life (MANSA), Brief Psychiatric Rating Scale (BPRS) for Schizophrenia and screening for suicidality; HbA1c, fatty acid profile, hsCRP, HOMA-IR, HOMA2-IR, advanced lipid testing, body weight, blood pressure, HR, waist circumference, body composition analysis	32% reduction in Brief Psychiatric Rating Scale, ↑proportion of participants who were in the recovery state at baseline *, ↑sleep quality *, improvement in cognition and mood, ↓anxiety, ↓depressive symptoms *, improvement in CGI scale, ↑life quality and satisfaction *, ↓body weight, waist circumference, systolic blood pressure, FMI, BMI *, ↓visceral adipose tissue, HbA1c, triglycerides, HOMA-IR *

F—females; M—males; KD—ketogenic diet; HbA1c—glycosylated hemoglobin; HOMA-IR—the homeostasis model assessment-estimated insulin resistance; hs-CRP—high sensitivity C-reactive protein; HR—heart rate; FMI—fat mass index; BMI—body mass index; * all participants (patients with SZ and BD analyzed as one group); I—intervention; C—control.

**Table 2:** Studies describing therapeutic effects of the ketogenic diet in depression.

Author(s), Year	Study Design	Number of Participants (Intervention/ Control)	Range Age of Participants (Years)	Dietary Intervention/ Assessment of Ketosis	Control Intervention	Duration	Outcome Measures	Main Findings
Cox et al., 2019 [83]	Case study	I: 1 (F)	65	KD, 65% fat, 25% protein, 10% carbohydrates with a time restricted feeding window; Nutritional education; High-intensity interval training; blood ketones pre/post intervention	none	12 weeks	The Patient Health Questionnaire 9 (PHQ-9), The General Self-Efficacy Scale (GSE), MetS Compliance Questionnaire (MSC), blood: HgA1C, glucose, ketones, HOMA-IR, the triglyceride/HDL cardiac risk ratio	Improvement in PHQ-9, GSE, and MSC scales; ↓HgA1C, glucose ketones, HOMA-IR, and triglycerides/HDL cardiac list ratio; ↓body weight; ↓amount of taken medications; ↑self-confidence, self-efficacy, energy, mood stability and cognition; sleep improvement
Pieklik et al., 2021 [74]	Case study	I: 1 (F)	21	KD, the Kalibra medical protocol diet; the urine ketone strip test	none	4 weeks	Body Image Questionnaire (KWCO), Scale of satisfaction with parts and parameters of the body, The Scale for the Using of Methods for Correcting Appearance, Scale of Perception of Peer Messages, Scale of Self Constructs and Beck Depression Inventory Scale (BDI)	↓body weight, mood stabilization, stabilization of daily rhythm, ↓anxiety, Improvement in BDI scale, a lack of suicidal thoughts
Danan et al., 2022 [82]	Retrospective analysis	I: 31; bipolar disorder type two (*n* = 13), schizoaffective disorder (*n* = 12), major depressive disorder (*n* = 7)	27–73	KD, <20 g (5%) carbohydrates per day, 15–20% protein, 75–80% fat; measurement of urine acetoacetate at least one time during the intervention period	none	6–248 days	Hamilton Depression Rating Scale (HAM-D), Montgomery–Åsberg Depression Rating Scale (MADRS), Positive and Negative Syndrome Scale (PANSS), Clinical Global Impressions Scale (CGI-S), metabolic health measures	improvement in HAM-D, MADRS and CGI-S scales; ↓the amount of taken medications; ↓body weight, blood pressure, blood glucose, and triglycerides

F—females; M—males; KD—ketogenic diet; HbA1c—glycated hemoglobin; I—intervention; C—control.

**Table 3:** Studies describing therapeutic effects of the ketogenic diet in bipolar disorder.

Author(s), Year	Study Design	Number of Participants (Intervention/ Control)	Range Age of Participants (Years)	Dietary Intervention/ Assessment of Ketosis	Control Intervention	Duration	Outcome Measures	Main Findings
Phelps et al., 2013 [114]	Two case studies	I: 2 (F)	69 and 30	KD, 8% carbohydrates, 22% protein, 70% fat (second case), the urine ketone strip test (first case)	none	2 years, 3 years	Patients’ and physicians’ observations	significant subjective reduction in symptoms, ↓the amount of taken medications, ↓depressive symptoms mood stabilization, ↑calm and confidence, comfort
Chmiel et al., 2022 [115]	Case study	I: 1 (M)	32	KD; 5% carbohydrate, 15% protein, 80% fat; ≥5040 kJ, <30 g carbohydrates per day; cyclic one-day fast introduced every 7–10 days, blood concentration of β-hydroxybutyrate	none	2 years	Body mass index (BMI), blood: CBC, lipid profile, glucose, liver tests, creatinine, uric acid	mood stabilization, elimination of anxiety, shorter and milder depressive states till complete remission, ↑mood, ↑energy, ↑cognitive functions and concentration, ↑periods of total remission of symptoms, ↓amount of taken medications, ↑HDL, ↓triglycerides
Needham et al., 2023 [113]	Pilot study	I: 27	26–54	A modified KD, 60–75% fat, 5–7% carbohydrates, additionally calories from protein, blood ketones	none	6–8 weeks	Medical and medication history, blood pressure and body mass index (BMI), Affective Lability Scale 18, Beck’s Depression Inventory, Young Mania Rating Scale, Within Trial Resource Use Questionnaire, EuroQol 5D quality of life instrument and the Work Productivity and Activity Impairment Questionnaire (tailored), Fasting venepuncture and MR brain scans, measurement of glucose and ketones on a KetoMojo device, daily ecological momentary assessments (EMAs) of anxiety, mood, energy, impulsivity and speed of thought, Visual Analogue Scale (VAS)	↓body weight, normalization of total cholesterol, LDL, and triglyceride levels EQ5D-5L at baseline and follow-up, respectively, were: mobility, 90 and 85%; self-care, 90 and 85%; usual activities, 65 and 55%; pain and discomfort, 45 and 45%; and anxiety and depression, 45 and 50% The visual analogue scale (VAS) utility scores at baseline and follow up were 66.7 and 64.2, ↓mean expenditure, ↑mean productivity loss
Sethi et al., 2024 [62]	Pilot study	I: 16	18–75	KD, 10% carbohydrate, 30% protein, 60% fat; ≥5040 kJ, <20 g carbohydrates per day; blood ketone meter at least once a week	none	4 months	Generalized Anxiety Disorder (GAD–7), Patient Health Questionnaire Depression Scale (PHQ–9), Pittsburgh Sleep Quality Index (PSQI), Clinical Mood Monitoring Forms (CMF), Clinical Global Impression–Schizophrenia (CGI–SCH) Scale, Global Assessment of Functioning (GAF), Manchester Short Assessment of Quality of Life (MANSA), Brief Psychiatric Rating Scale (BPRS) for Schizophrenia and screening for suicidality; HbA1c, fatty acid profile, hsCRP, HOMA-IR, HOMA2-IR, advanced lipid testing, body weight, blood pressure, HR, waist circumference, body composition analysis	improvement in CGI scale: severity of mental illness showed improvement of >1 point in 69% of participants, ↑proportion of participants who were in the recovery state at baseline *, ↑sleep quality *, ↓anxiety *, ↓depressive symptoms, ↑life quality and satisfaction *, ↓body weight, waist and circumference and systolic blood pressure and FMI and BMI * ↓visceral adipose tissue, HbA1c, triglycerides, HOMA-IR *

F—females; M—males; KD—ketogenic diet; HbA1c—glycosylated hemoglobin; HOMA-IR—the homeostasis model assessment-estimated insulin resistance; hs-CRP—high sensitivity C—reactive protein; HR—heart rate; FMI—fat mass index; BMI—body mass index; * all participants (patients with SZ and BD analyzed as one group); I—intervention; C—control.

**Table 4:** Ongoing clinical trials exploring the role of the KD in psychiatric disorders.

Identifier	Study Title	Status	Locations	Conditions	Hospitalized/ Amulatory Patients	Age of Participants	Enrollment	Intervention	Control	Timeframe	Primary Outcome and Timeframe
NCT03873922 [118]	Dietary Intervention for Psychotic Disorders: a Pilot Intervention Study of Ketogenic Diet for Psychotic Symptoms—PsyDiet Pilot Study	Recruiting	Kuopio, Finland	Patients with psychotic symptoms (ICD-10 diagnosis F20-29)	Hospitalized	≥18	40	Ketogenic diet (15–20 g CHO/d)	Conventional hospital meals	6 weeks	Changes in PANSS and diet feasibility
NCT05968638 [119]	Single-Blind Randomized Ketogenic Diet vs. Control Diet in People With Schizophrenia	Recruiting	Catonsville, Maryland, United States	Schizophrenia/Schizoaffective disorder (DSM-IV/DSM-5)	N/A	18–64	50	Ketogenic diet	Standard diet	3 months	Changes in BPRS
NCT05268809 [120]	Can Neural Network Instability in Schizophrenia be Improved With a Very Low Carbohydrate Ketogenic Diet?	Recruiting	San Francisco, California, United States	Schizophrenia/Schizoaffective disorder/Bipolar disorder (SCID-5)	Ambulatory	18–65	70	Ketogenic diet (70% F; 10% CHO; 20% PRO); 3 meals + snak/d	The diet as usual	4 weeks	Changes in network stabilization, cognition, waist to hip ratio, HOMA-IR, blood: CRP
NCT06221852 [121]	A Randomized Controlled Clinical Trial of Ketogenic and Nutritional Interventions for Brain Energy Metabolism and Psychiatric Symptoms in First Episode Bipolar Disorder	Not yet recruiting	Belmont, Massachusetts, United States	Bipolar disorder/Schizoaffective disorder, onset of illness in the last 7 years (DSM-5)	N/A	18–45	50	Ketogenic diet (75–80% F; 7% CHO; 13–18% PRO); 3 meals + snak/d; normocaloric	Dietary Guidelines for Americans; 3 meals + snak/d; normocaloric	12 weeks	Changes in PANSS, HAM-D, YMRS, CGI, insulin resistance, brain NAD+/NADH ratio and creatine kinase forward reaction rate
NCT06081426 [122]	Elucidating Neurobiological Mechanisms Underlying the Therapeutic Effect of the Ketogenic Diet in Bipolar Disorder (BD): a Multidisciplinary Mechanistic Study	Recruiting	Pittsburgh, Pennsylvania, United States	Bipolar disorder I/Bipolar disorder II, hypomanic/euthymic (DSM-5)	N/A	18–30	107	Ketogenic diet	Non-ketogenic diet/No diet	8–10 weeks	Changes in YMRS, brain activity and connectivity, brain concentration of GABA, glutamate, lactate, blood: glucose, lipids, bilirubin, total protein, albumin, liver enzymes
NCT05705063 [123]	Impact of A Low-Carbohydrate, High-Fat, Ketogenic Diet on Obesity, Metabolic Abnormalities, and Psychiatric Symptoms on Patients With Bipolar Disorder (BPD)	Not yet recruiting	Stanford, California, United States	Bipolar disorder (DSM-5)	N/A	18–75	30	Ketogenic diet	No control group	6 weeks	Changes in weight, waist circumference, visceral fat mass, body fat mass, heart rate, blood pressure, HOMA-IR, blood: HbA1c, hs-CRP, lipids
NCT06105762 [124]	KETO-MOOD: Ketogenic Diet for Microbiome Optimization and Overcoming Depression	Not yet recruiting	Basel, Switzerland	Major Depressive Disorder/Bipolar Depression (ICD-10/ICD-11)	N/A	18–70	120	Ketogenic diet (MAD, <20 g CHO/d)	Mixed diet following the recommendations for healthy nutrition by the Schweizerische Gesellschaft für Ernährung (Société Suisse de Nutrition) (45–60% CHO)	8 weeks	Changes in HAM-D17
NCT05558995 [85]	Effects and Mechanistic Aspects of Ketogenic Diet in Individuals With Major Depressive Disorder: A Pilot Study	Recruiting	Kingston, Ontario, Canada	Major Depressive Disorder (DSM-5)	Ambulatory	18–55	10	Ketogenic diet (20–30 g CHO/d; 80–100 g PRO/d; PUFA; MUFA) + vitamins, minerals in caps	No control group	12 weeks	Adherence to diet
NCT06091163 [125]	A Randomised Controlled Trial Evaluating the Efficacy and Mechanisms of a Ketogenic Diet as an Adjunctive Treatment for People With Treatment-resistant Depression	Recruiting	Oxford, United Kingdom	Depression	Ambulatory	18–65	100	Ketogenic diet (20–50 g CHO estimated on a 2000 kcal/d); 3 meals + snacks	Modified fat and phytonutrient diet	6 weeks	Changes in PHQ-9
NCT06080932 [126]	Ketogenic Intervention in Depression	Recruiting	Columbus, Ohio, United States	Major Depressive Disorder (DSM-5)	Ambulatory	18–30	30	Ketogenic diet (<50 g CHO/d, ~1.5 g PRO/kg reference weight)	No control group	~up to 12 weeks	Changes in HAM-D17, WHO-5, blood: ketones and glucose

DSM—Diagnostic and Statistical Manual of Mental Disorders; ICD—International Classification of Diseases; SCID—The Structured Clinical Interview for DSM; N/A—Not Applicable; CHO—carbohydrates; PRO—protein; PANSS—The Positive and Negative Syndrome Scale; BPRS—The Brief Psychiatric Rating Scale; HAM-D—Hamilton Depression Rating Scale; YMRS—Young Mania Rating Scale; PHQ—Patient Health Questionnaire; CGI—The Clinical Global Impressions Scale; WHO-5—The World Health Organisation-Five Well-Being Index; HOMA-IR—Homeostatic Model Assessment for Insulin Resistance; HbA1c—Glycated hemoglobin; CRP—C-Reactive Protein; GABA—Gamma Amino Butyric Acid; NAD/NADH—the ratio of oxidized and reduced forms of nicotinamide adenine dinucleotide.

**Table 5:** A practical guide to implementing the ketogenic diet in psychiatric disorders [156].

Potential Risk	Risk Group	Advice
Dyslipidemia	Patients treated with atypical antipsychotic medication. Overweight or obese individuals.	Replace foods with high SFA and trans unsaturated fatty acid with sources of PUFA and MUFA. Less: meat with high-fat content, lard, butter, eggs, coconut/palm oil More: olive oil, rapeseed oil, flax oil, avocado, fatty fish, allowed amounts of nuts and seeds Supplementation of omega-3 fatty acids (>1 g EPA or EPA/DHA ratio 1:1) could be helpful
Constipation	Patients treated with medication with anticholinergic effects (clozapine, olanzapine, phenothiazine derivatives, TCA72).	Choose vegetables, nuts, and seeds with more fiber and less carbohydrate content; the examples of the ratio of fiber to carbohydrate in some foods are presented below: Spinach 1:1.15 Sesame 1:1.27 Desiccated coconut 1:1.28 Mushrooms 1:1.3 Chives 1:1.56 Celery tuber 1:1.57 Almonds 1:1.59 Kale 1:1.6 Brussels sprout 1:1.6 Hazelnut 1:1.67 Chinese cabbage 1:1.68 Horseradish 1:1.76 Green beans 1:1.95 Lettuce 1:2.07 Broccoli 1:2.08 Cauliflower 1:2.08 Parsley, root 1:2.14 Peanuts 1:2.63 Red cabbage 1:2.68 Walnuts 1:2.77 Tomatoes 1:3 Zucchini 1:3.2 Red pepper 1:3.3 Cucumber, pickled 1:3.8 Onion 1:4.05 Cucumber 1:5.8 *
Calcium deficiency, osteoporosis risk	Patients treated with SSRI, valproic acid and with high prolactin levels after pharmacotherapy.	Supplementation of vitamin D according to guidelines. Monitoring vitamin D status. Intake of foods rich in Ca allowed on KD, such as: cheese (hard cheese, feta, mozzarella), Greek yoghurt, cream, or vegetables with a high ratio of Ca to P. The ratio of Ca to P in some foods is presented below; 1:1 or higher Ca is recommended. Kale 1:0.36 Parsley, leaves 1:0.44 Cabbage, white 1:0.49 Cheese, Emmentaler 1:0.5 Sauerkraut 1:0.5 Chives 1:0.54 Onion 1:0.56 Brussels, sprout 1:0.58 Cheese, parmesan 1:0.59 Cheese, brie 1:0.63 Pumpkin 1:0.65 Cabbage, red 1:0.67 Cheese, camembert 1:0.8 Cabbage, Chinese 1:0.82 Broccoli 1:1.38 Cucumber, pickled 1:1.5 Cucumber 1:1.53 Almonds 1:1.9 Cauliflower 1:2.15 Walnuts 1:3.82 Sunflower seeds 1:5.98 Peanuts 1:6.64 Egg, white 1:8.5 Cod 1:92 Roast beef 1:95.5 Chicken, leg 1:98 Egg 1:102 Breast, chicken 1:120 Salmon 1:133 Egg, yolk 1:293 **

TCA—tricyclic antidepressants; SSRI—selective serotonin reuptake inhibitor; SFA—saturated fatty acid PUFA—polyunsaturated fatty acids; MUFA—monounsaturated fatty acids; EPA—eicosapentaenoic acid; DHA—docosahexaenoic acid; Ca—calcium; P—phosphorus; KD—ketogenic diet; * fiber to carbohydrate ratio; ** Ca to P ratio.

## Data Availability

Not applicable.
